# Visualization of scientific production in *Caenorhabditis elegans*: a bibliometric analysis (1980–2023)

**DOI:** 10.1186/s44342-024-00002-7

**Published:** 2024-05-31

**Authors:** Şeyda Berk, Serkan Özdemir, Ayşe Nur Pektaş

**Affiliations:** 1 Department of Molecular Biology and Genetics, Faculty of Science, Sivas Cumhuriyet University, Sivas, 58140 Turkey; 2 Department of Forestry, Isparta University of Applied Sciences, Isparta, 32260 Turkey; 3 Advanced Technology Research and Application Center (CUTAM), Sivas Cumhuriyet University, Sivas, 58140 Turkey

**Keywords:** Bibliometric, Biblioshiny, *C. elegans*, Nematode, Web of Science

## Abstract

*Caenorhabditis elegans* (*C. elegans*) is a nematode and model organism whose entire genome has been mapped, which allows for easy observation of the organism’s development due to its transparent structure, and which is appealing due to its ease of crossover, ease of culture, and low cost. Despite being separated by nearly a billion years of evolution, *C. elegans* homologs have been identified for the vast majority of human genes and are associated with *C. elegans* for many biological processes such as apoptosis, cell signaling, cell cycle, cell polarity, metabolism, and aging. A detailed bibliometric study is performed here to examine publication trends in this field. Data were taken from the Web of Science database and analyzed using the bibliometric application Biblioshiny (RStudio). In terms of publication, the results indicated a gradual increase each year between 1980 and 2023. A total of 20,322 records were issued in 96 countries, the majority of which were in the USA, China, and Japan. The most prolific writers, the journals most engaged in the area, the nations, institutions, and keywords used by authors were all determined using the Web of Science database and bibliometric rules. The number of papers in the *C. elegans* research field is increasing exponentially, and *Genetics* is the journal with the highest number of articles. This study presents how research patterns have evolved throughout time. As a result, worldwide cooperation and a potential field can be developed.

## Introduction

The multicellular organism *C. elegans*, with its fully known genetic profile, is a classic model animal with great contributions to life and environmental sciences [1]. *C. elegans* are small (about 1 mm long), soil-dwelling, bacteria-eating nematodes that can occur as self-pollinated hermaphrodites or males [1, 2]. Males represent a minority of the population (approximately 0.2%). Healthy hermaphrodites produce up to 300 offspring on their own, but can produce about 1000 offspring when mated with a male [3]. The nematode *C. elegans* is a well-studied organism with a 3-day generation time, making it valuable for studying effects that could last for several generations [4]. For experimental research at the metabolic and genomic levels in vivo throughout the past few decades, *C. elegans* has served as a model system to replicate the majority of human disorders [5, 6]. Furthermore, because of its many benefits, such as easy genetic control, a short lifetime, and clear age-related physiological changes, this organism is a very effective model for aging research [7]. Moreover, *C. elegans* aging research has generated promising results in identifying molecular signals, epigenetic modifications, and transcriptional regulators related with longevity, extending our understanding of how organisms age [8]. Anatomical and functional characteristics of *C. elegans* deteriorate with age, including motility, tissue integrity, immunity, learning, and memory. Many age-related alterations in the expression of microRNAs and stress-responsive genes, as well as RNA and protein quality control mechanisms, have been observed in *C. elegans*. Many of these aging-related alterations provide information about animal health and can be used as biomarkers in aging research [9]. In addition to all of its advantages, nematode trials raise no ethical problems [10]. The nematode *C. elegans* has been useful in several areas of pharmaceutical and scientific research since the early 1960s [11–15]. In addition, *C. elegans* research has also contributed to key advances in the life sciences, such as the identification of genetic regulators of programmed cell death, the use of green fluorescent protein (GFP) as a protein marker, and the discovery of RNA interference [16–18].

Bibliometric studies the quantitative aspects of science information [19]. This type of analysis is considered a susceptible method for evaluating research results based on statistical tools and for studying the metrological properties of information produced in a particular field. [20]. Our rationale for a bibliometric study is the considerable amount of information about *C. elegans* accumulated in several databases. Bibliometric studies are conducted to assess the quantity and development of scientific output among countries, institutions, research units, and scholars in key subject areas. The bibliographic literature has experienced a huge boom in recent decades, driven by digitization, information systematization, and the creation of numerous and diverse databases of scientific literature that are electronically accessible around the world [19]. This method is considered a useful tool to aid decision-making in setting research priorities, tracking scientific and technological developments, allocating funds, and recognizing scientific excellence [21].

A bibliometric research evaluates annual publications, the most prolific and notable authors, significant institutions and nations, or the most cited articles to assess the scientific significance and progress of a specific field of knowledge. This will be the first study to look at scientific literature about *C. elegans*. It will provide a comprehensive view of the scientific community, made feasible by a bibliometric technique that analyzes data and metadata from previously published works on the subject. As a result, the primary goals are to examine the annual publication trend, identify the most prolific and referenced journals and authors, and highlight the most frequently used keywords and articles.

## Methods

### Source of data

**Fig. 1:**
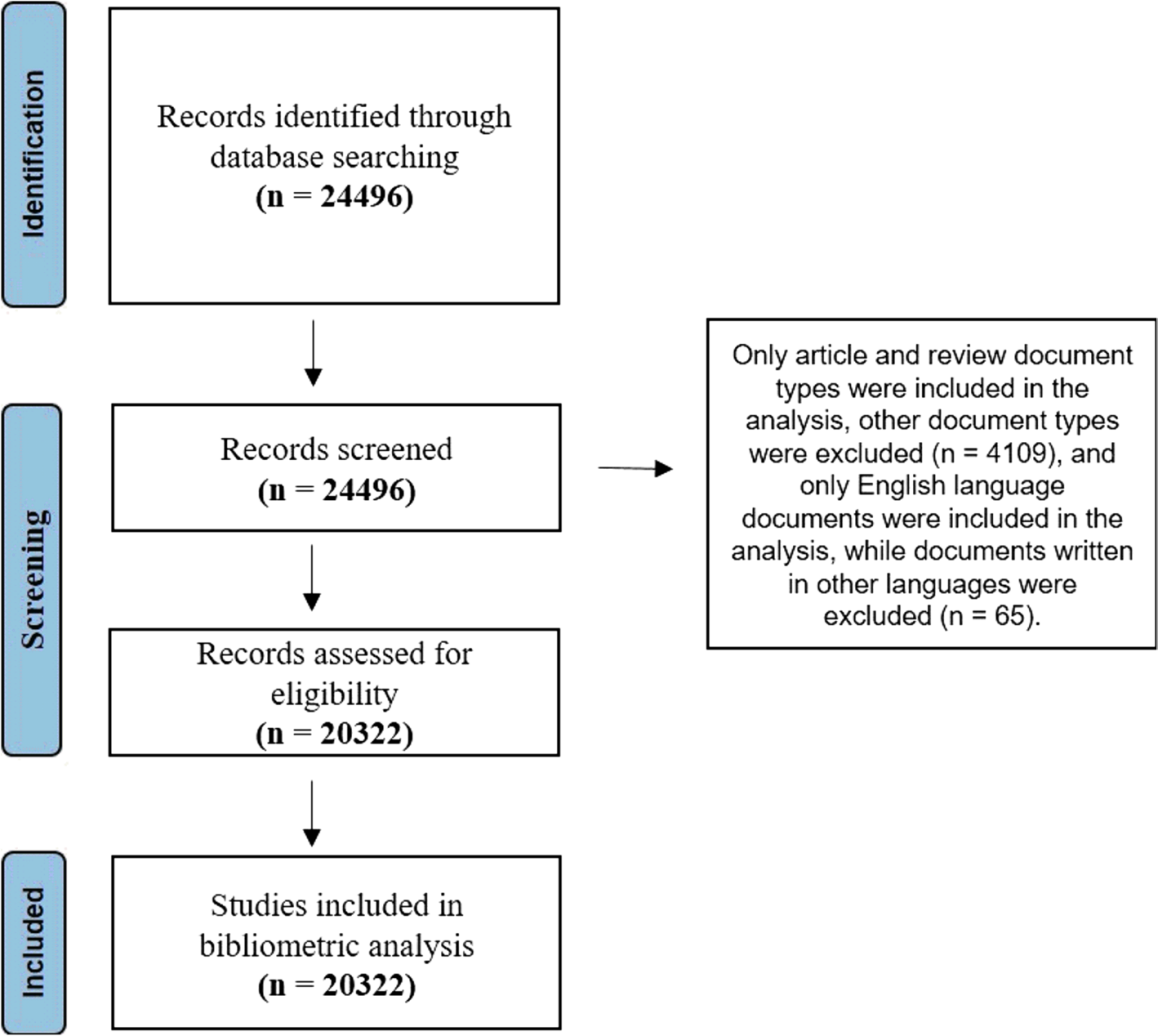
PRISMA flow diagram

The published academic works on *C. elegans* included in the Web of Science (WOS) core collection database were studied in this bibliometric analysis. WOS is the most trusted global citation database, with over 21,000 peer-reviewed journals, and the most often used for academic paper analysis [22]. A comprehensive four-step approach was framed in this study as shown in Fig. 1. Between the years 1980 and 2023 (31st December), a total of 24,496 documents were extracted related to “*C. elegans*” or “*Caenorhabditis elegans*.” Subsequently, the document types were filtered by selecting the “articles” and “reviews,” and the language was restricted to English. Ultimately, 20,322 studies were refined. All data were collected the same day from the WOSCC to avoid bias caused by database updates.

### Statistical analysis

In the present study, Biblioshiny [23, 24] is utilized for bibliometric analyses. Biblioshiny is a free, web-based interface that operates with the R operating system and works in conjunction with the open-source R software [25]. To perform the analyses by connecting to the web interface through R, the required package for bibliometric analysis [26] was first installed using the following commands in sequence: install.packages (“devtools”) and devtools::install_github (massimoaria/bibliometrix). Subsequently, the library of the package was activated with the command library (bibliometrix). Finally, the database was accessed using the code biblioshiny (maxUploadSize = 500). Here, the parameter “maxUploadSize = 500” was used to increase the upper limit of the WOSCC text file size that can be uploaded to the database, as the default setting was insufficient due to the download of 20,322 documents from the WOSCC database. Therefore, the mentioned code was used to raise this limit.

Within the Biblioshiny database, the WOS option was selected as the database choice, and a text file prepared for 20,322 documents was imported. Then, bibliometric analyses were conducted using the Overview, Sources, Authors, Documents, Conceptual Structure, Intellectual Structure, and Social Structure tabs. In the analyses, the annual production of the selected reference topics was initially examined, and the percentages of the top 10 most frequently used keywords were determined. Subsequently, an analysis approach from general to specific was adopted, and bibliometric analyses were carried out for countries, affiliations, journals, and authors, respectively.

## Results and discussion

### Exponential growth in annual publications

**Fig. 2:**
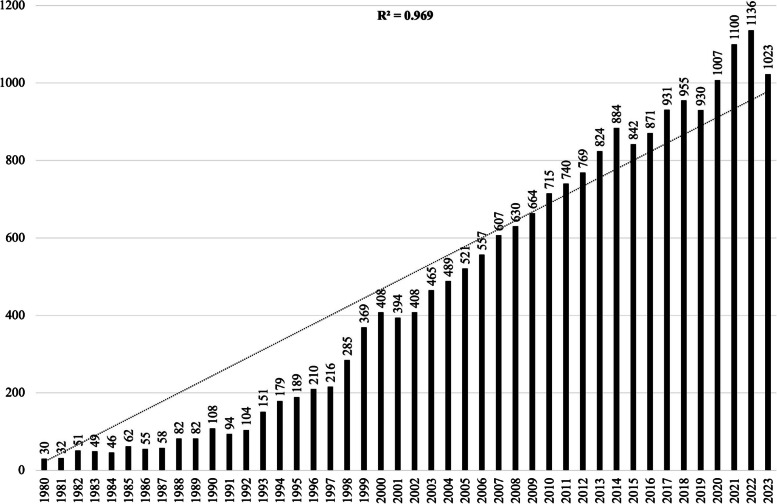
Annual production of research on *C. elegans* from 1980 to 2023 by the online bibliometric analysis

As a result of the analysis carried out on 3 January 2024, a total of 24,496 documents were obtained in line with the appropriate keywords. As a result of subsequent exclusions, 20,322 articles and review articles published between 1980 and 2023 were obtained. The annual number of publications is shown in Fig. 2. According to this graph, it was seen that there were 30 publications in 1980. Afterwards, we observed that the number of articles gradually increased from year to year. The year 2021 and 2022 has the highest rate with 1100 and 1136 publications, respectively. As a result of our analysis, it reached 1023 publications in 2023 (Fig. 2). In addition, the development-growth trend model (*R*^2^ = 0.969) estimates the number of studies on *C. elegans*.

### Research area

**Fig. 3:**
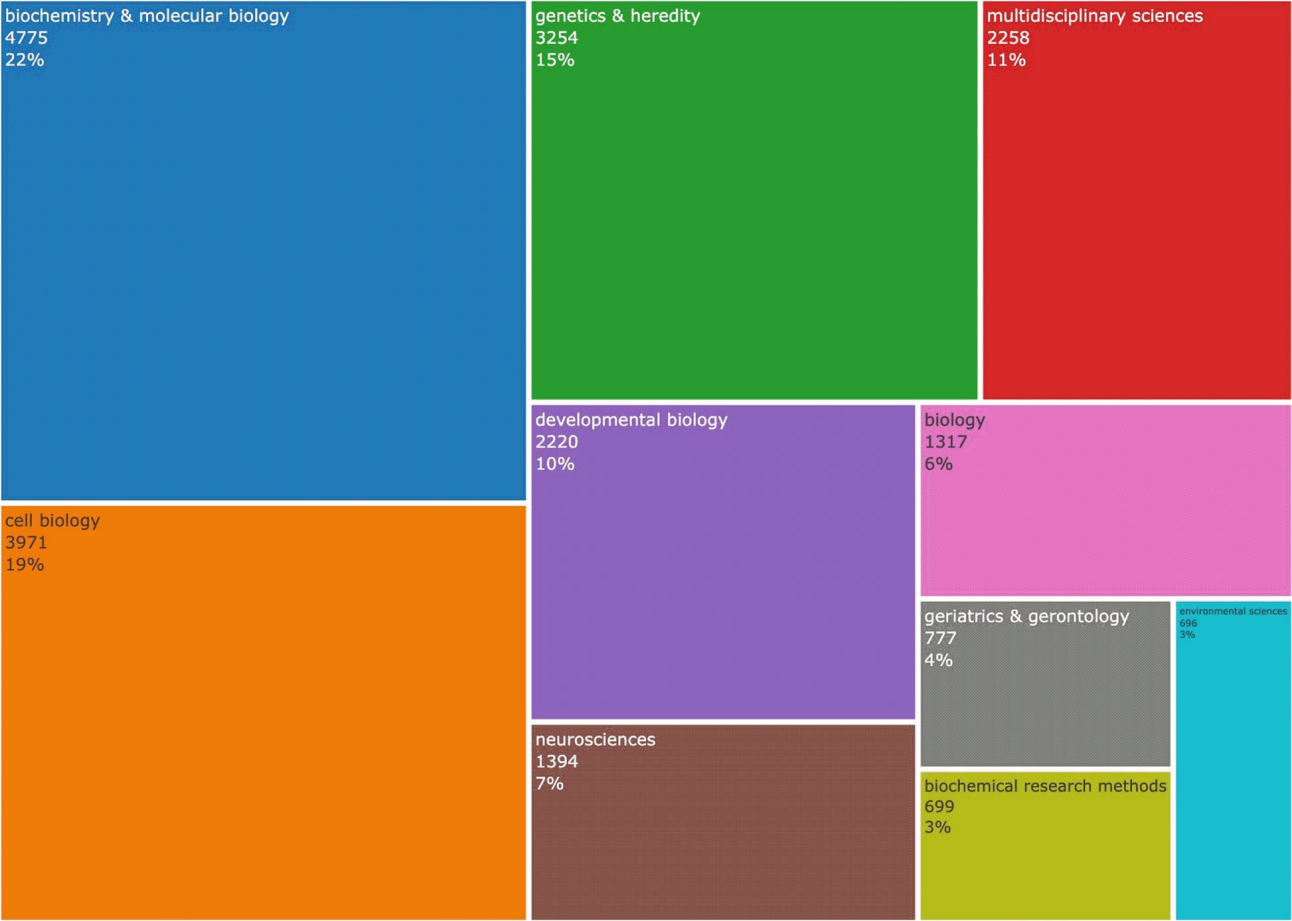
TreeMap of the top 10 subject categories

Disciplinary categories (Web of Science Categories) were extracted from the results, as presented on the TreeMap in Fig. 3. Biochemistry and Molecular Biology (22%), Cell Biology (19%), Genetics and Heredity (15%), Multidisciplinary Sciences (11%), and Developmental Biology (10%) are the most prominent categories. Together, these five categories account for 77% of all research.

### Countries/regions and organization

**Fig. 4:**
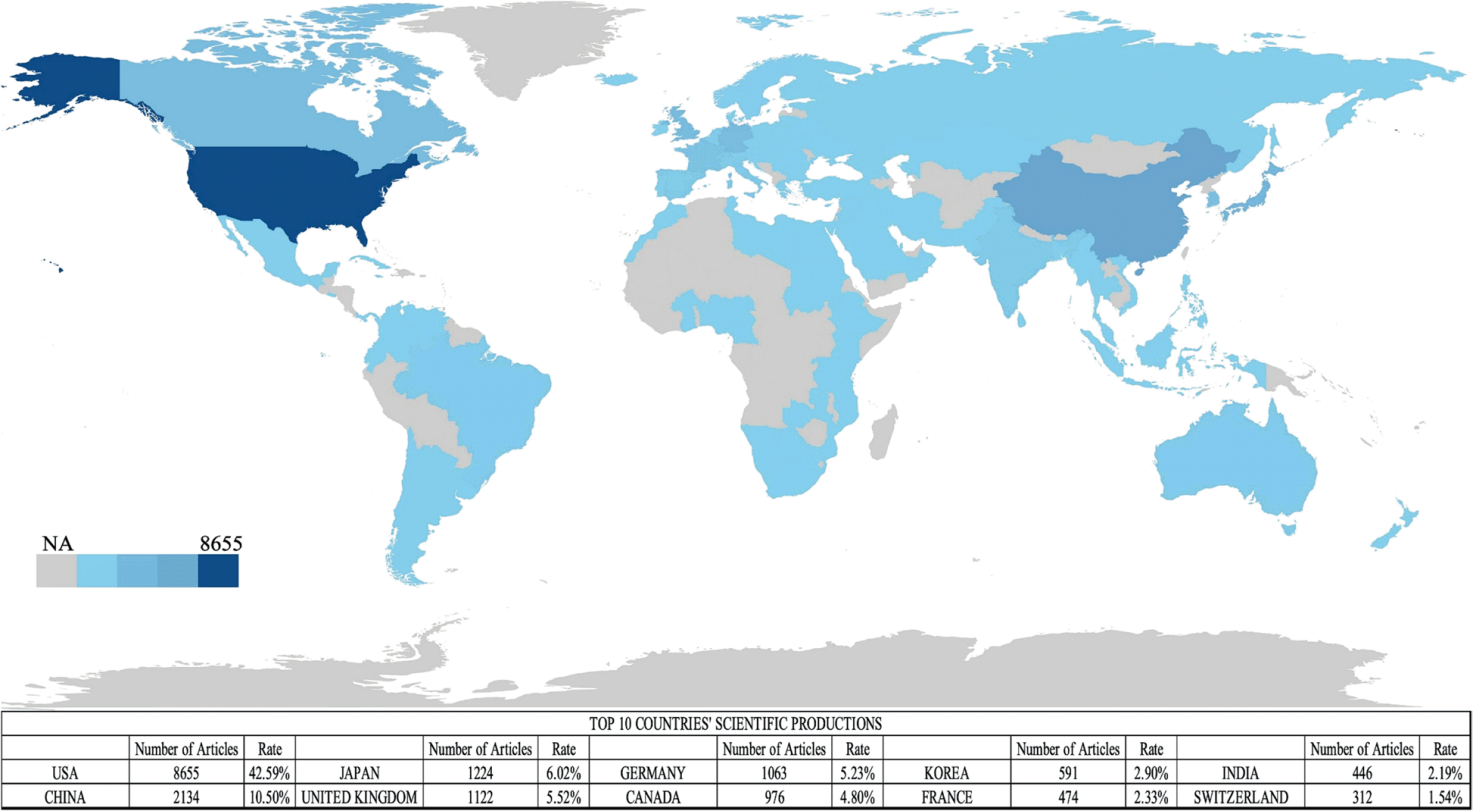
Scientific productions of top 10 countries on *C. elegans*. The map was created using the “Biblioshiny” program. Different shades of blue signify different levels of productivity: dark blue indicates high productivity; gray indicates no articles

Between 1980 and 2023, researchers connected with institutions in 96 countries co-authored a total of 20,322 articles on *C. elegans*. The USA (8655), China (2134), Japan (1224), the UK (1122), Germany (1063), Canada (976), South Korea (591), France (474), India (446), and Switzerland (312) had the most co-authored papers overall (Fig. 4).

**Fig. 5:**
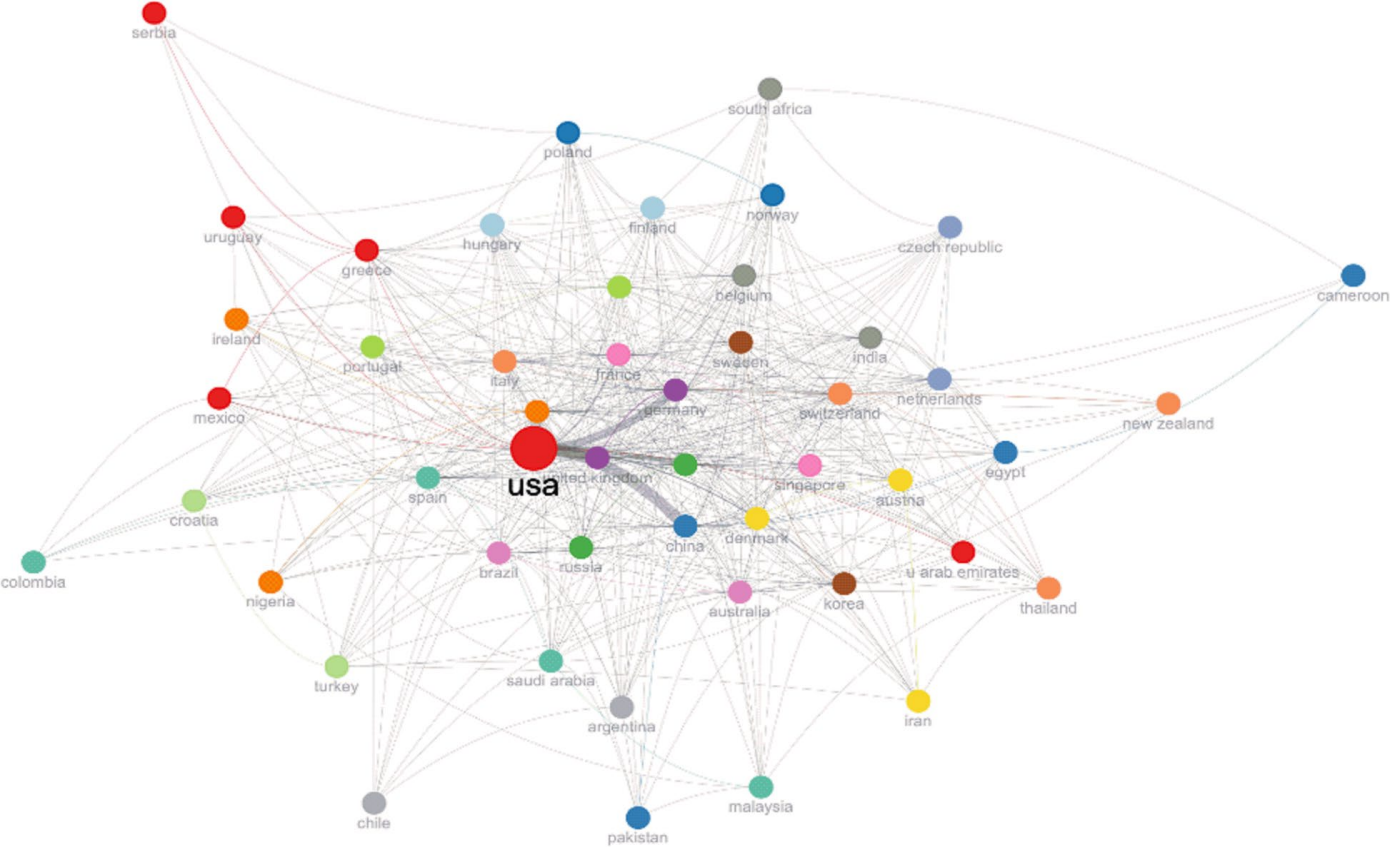
Bibliometric map of the global collaboration network of countries for *C. elegans* research from 1980 to 2023

A map of the co-authorship analysis is shown in Fig. 5 and reveals networks of collaboration between nations working on *C. elegans* between 1980 and 2023. The USA is the largest node on the map out of all the countries depicted in Fig. 5. The USA and Germany have also conducted the most (64) international research collaborations, followed by the UK (50), China (49), and Japan (42) (Table 1). According to the pagerank values obtained as a consequence of our investigation, the countries shown in Fig. 5 have significant cooperation networks because nearly all of them are connected in the network. Countries in the map’s extremes and with the lowest pagerank values (Serbia, Colombia, and Cameroon) have been less productive in this regard, resulting in weaker international cooperation networks. The strongest collaborators of the USA have been with China, UK, Canada, Germany, and Japan (Fig. 5). This country has also co-authored several publications with some European countries (such as Switzerland, France, The Netherlands, Australia and Spain) and fewer collaborations with Belarus, Iceland, Libya, Luxembourg, Malta, Oman, Panama, Slovenia, Tanzania, and Ukraine.

**Table 1:** Top 10 countries on *C. elegans*

Rank	Country	Documents	Total citation	Link
1	USA	8655	549,639	64
2	China	2134	35,937	49
3	Japan	1224	35,471	42
4	UK	1122	67,243	50
5	Germany	1063	38,526	64
6	Canada	976	33,945	39
7	S. Korea	591	11,954	21
8	France	474	17,996	36
9	India	446	6288	24
10	Switzerland	312	12,129	30

**Fig. 6:**
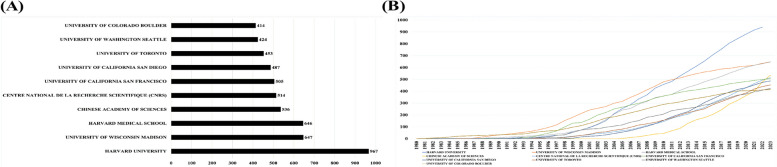
**A** Top 10 most relevant affiliations. **B** Year-wise publications of the top 10 most relevant affiliations

**Fig. 7:**
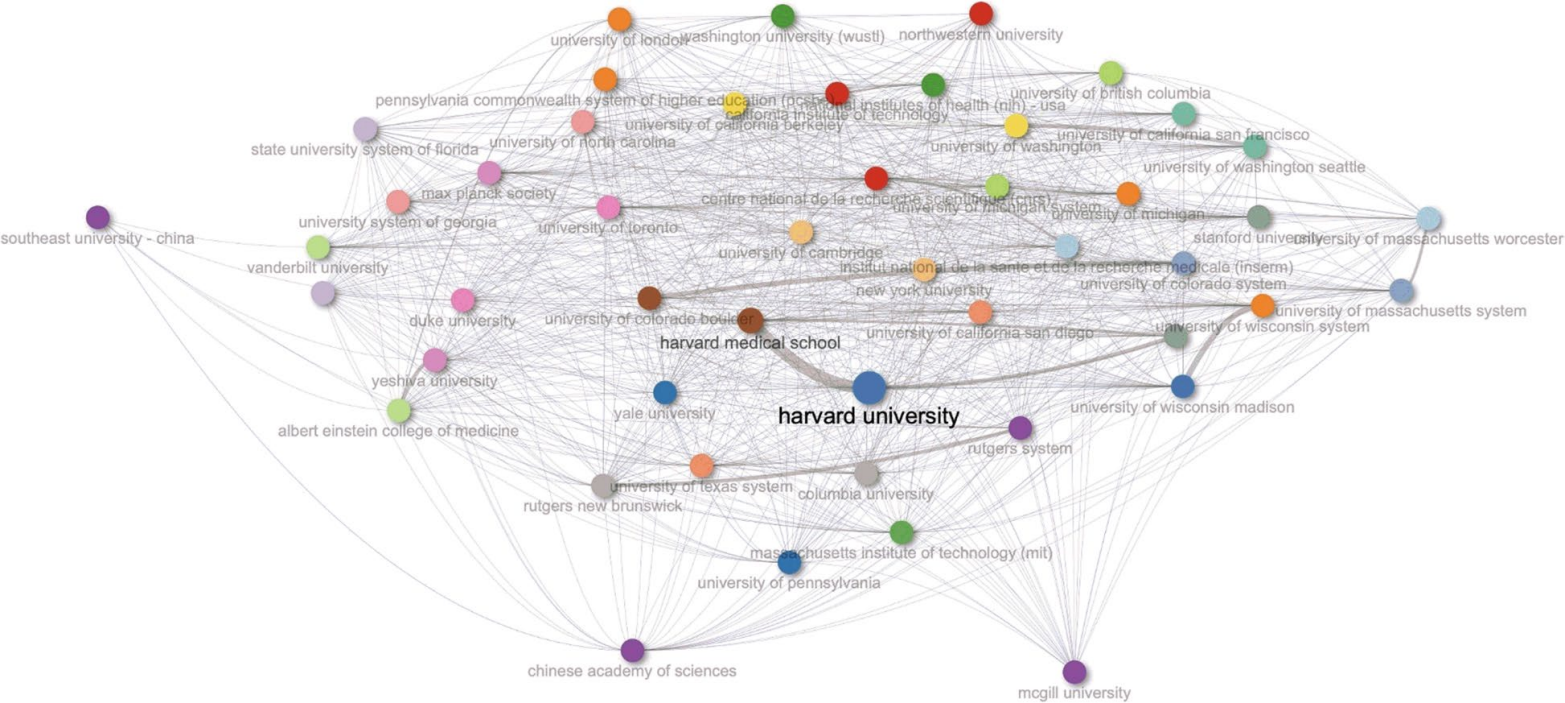
Bibliometric map of the global collaboration network of institutions for *C. elegans* research from 1980 to 2023

The top ten most active organizations studying on *C. elegans* are shown in Fig. 6. Harvard University, which published the highest number of related articles, had 967 related articles (Fig. 6A). In addition, looking at the distribution of publications by years, an exponential increase was observed in the ten most active institutions, and the highest increase belongs to Harvard University (Fig. 6B). As shown in Fig. 6, seven of the 10 large institutions were from the US and the other three institutions from Canada, China, and France. Harvard University (USA, 967), University of Wisconsin Madison (USA, 647), and Harvard Medical School (USA, 536) ranked first through third in terms of the number of articles published. As seen in Fig. 7, some articles were completed in collaboration with multiple institutions. The data show that *C. elegans* research is mostly USA-based and universities in the USA cooperate more with each other and show that other countries should improve their cooperation with their counterparts.

### Journals analysis

Between 1980 and 2023, 2147 journals and books published research articles and reviews on *C. elegans*. Table 2 displays the top 20 journals, which published approximately 33.59% of the documents (7077/20,322). *Genetics* was the most dynamic journal-related topic with *C. elegans*, followed by *Development*, *Developmental Biology*, *PLOS One*, *Proceedings of The National Academy of Sciences of the United States of America*, *PLOS Genetics*, *Journal of Biological Chemistry*, *Current Biology*, *G3-Genes Genomes Genetics*, and *Journal of Neuroscience*. However, when the citation numbers for the articles of each journal are examined, as seen in Table 2, *Nature* ranks first (212 articles, 964,876 citations), and the most prominent publications are as follows: *PLOS One*, Proc*eedings of The National Academy of Sciences of the United States of America*, and *Scientific Reports*. In general, as given in Table 2, the journals in the top 20 are journals with multidisciplinary interests. However, *Development*, *Developmental Biology*, *Journal of Neuroscience*, *Genes and Development*, and *Aging Cell* journals include many studies focused on *C. elegans*, especially in the fields of aging and developmental biology.

**Table 2:** Top 20 journals with the largest number of publications

Rank	Journals	Documents	Citation	Impact factor (IF):	Journal citation indicator (JCI):	JCR
1	*Genetics*	937	46,415	3.3	1	Q2
2	*Development*	681	65,994	4.6	1.46	Q1
3	*Developmental Biology*	608	31,108	2.7	0.79	Q2
4	*PLOS One*	570	944,409	3.7	0.91	Q1
5	*Proceedings of the National Academy of Sciences of the United States of America*	505	788,686	11.1	2.51	Q1
6	*PLOS Genetics*	437	56,584	4.5	1.34	Q1
7	*Journal OF Biological Chemistry*	345	392,757	4.8	0.91	Q2
8	*Current Biology*	338	85,124	9.2	1.83	Q1
9	*G3-Genes Genomes Genetics*	267	9935	2.6	0.8	Q2
10	*Journal of Neuroscience*	244	192,643	5.3	1.48	Q1
11	*Scientific Reports*	244	696,320	4.6	1.05	Q2
12	*eLife*	242	89,502	7.7	2.21	Q1
13	*Genes & Development*	239	54,132	10.5	2.04	Q1
14	*Cell*	238	338,069	64.5	9.55	Q1
15	*Molecular Biology of the Cell*	226	33,861	3.3	0.54	Q3
16	*Biochemical and Biophysical Research Communications*	218	108,401	3.1	0.76	Q3
17	*Nature*	212	964,876	64.8	11.32	Q1
18	*JoVE-Journal of Visualized Experiments*	180	24,901	1.2	0.28	Q3
19	*Aging Cell*	174	16,926	7.8	1.68	Q1
20	*Journal of Cell Biology*	172	76,617	7.8	1.26	Q1

**Fig. 8:**
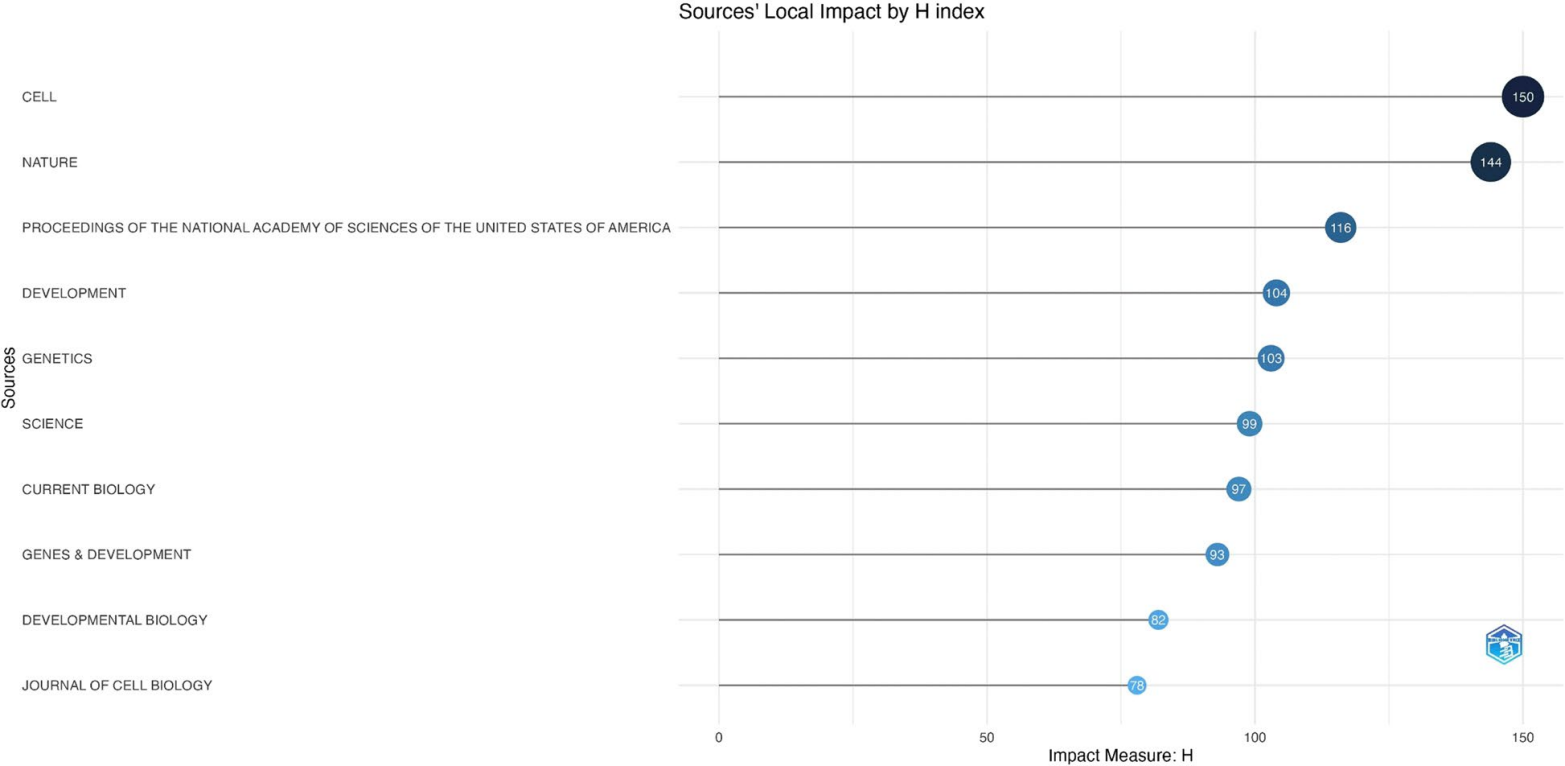
Top 10 journal ranking according to h-index values

The impact factor (IF) of 20 journals was from 1.2 to 64.8, and *Nature* had a maximum IF of 64.8 (Q1), and *Jove-Journal of Visualized Experiments* had a minimum IF of 1.2 (Q3) (Table 2). The number of journals dealing with *C. elegans* research was relatively wide and spread out between Q1 and Q3. The two journals with the greatest h-indexes are *Cell* and *Nature*, with scores of 150 and 144, respectively, as shown in Fig. 8. Considering the journals according to the IF and JCR values in Table 2, *Cell* and *Nature* may be the most popular journals for *C. elegans* research.

### Authors analysis

**Fig. 9:**
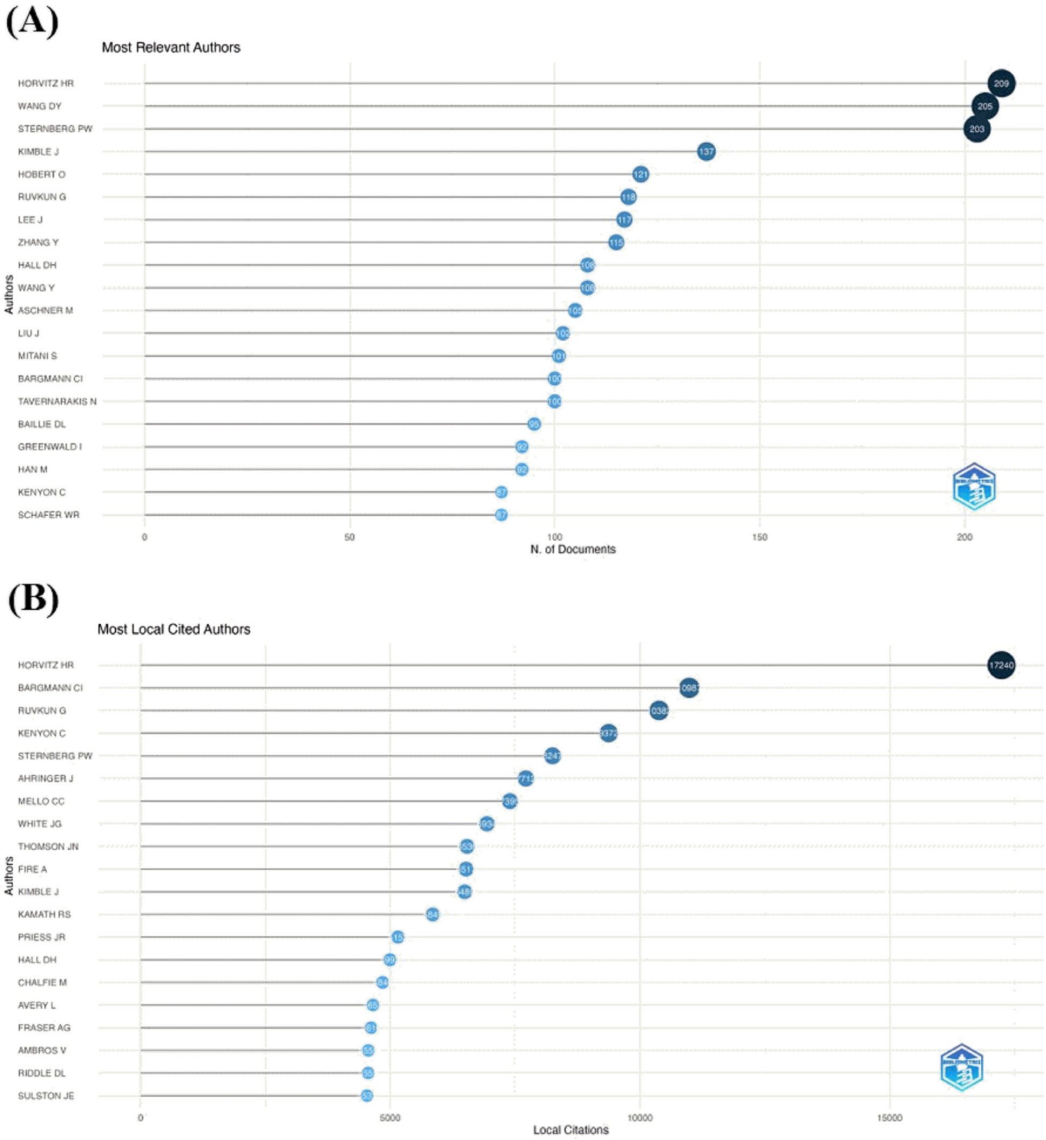
Top 20 active authors with most documents (**A**) and citations (**B**)

A total of 41,346 authors drafted the 20,322 documents on *C. elegans*. Several methodologies were utilized to identify the most influential researchers in this discipline, as well as the number of articles published, citations, and collaborations. The most active author in this field was Horvitz H.R. (Massachusetts Institute of Technology), with 209 documents, followed by Wang D.Y. (Southeast University), Sternberg P.W. (California Institute of Technology), Kimble J. (University of Wisconsin-Madison), and Hobert O. (Columbia University), as shown in Fig. 9A. Furthermore, Horvitz H.R. had the most citations in this ranking (17,240), followed by Bargmann C.I. (10,987), Ruvkun G. (10,382), Kenjon J. (9372), and Sternberg P.W. (8247) (Fig. 9B).

Lotka’s law found that the most prolific authors were 49 co-authors with five or more papers (international co-authorships 22.44%). Several important groups of co-authors were identified in this field; the most notable group of researchers was led by Horvitz, who collaborated with other writers. Hortvitz is recognized as the most significant author of *C. elegans* in the current analysis (from 1980 to 2023), leading the categories for the number of author partnerships. In addition, he is a co-author of the article “Genetic control of programmed cell death in the nematode *C. elegans*” [27] which was published in 1986 and has been cited 2486 times as of this article. He also shared the 2002 Nobel Prize in Physiology or Medicine for identifying the genes that drive cell death in the nematode worm *C. elegans*.

**Fig. 10:**
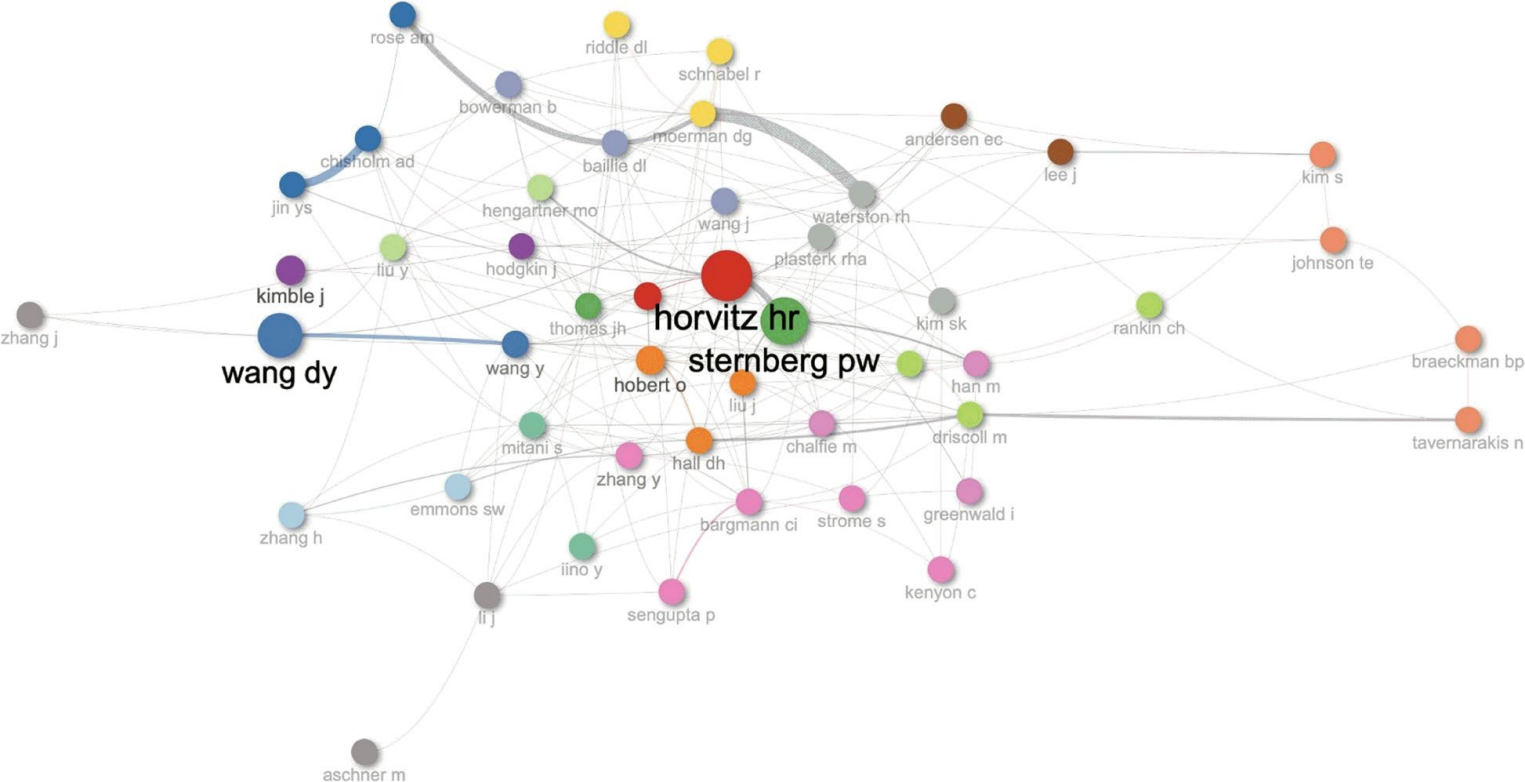
*C. elegans* collaboration network map

The most important co-authors and their network of collaborations are displayed in Fig. 10. There were various cooperation groupings among the authors. According to the number of publications and the number of authors that collaborated, Horvitz H.R., Wang D.Y., and Sternberg P.W. had the most central nodes in Fig. 10. The social structure of the field’s authors was also examined through social structure analysis. We have shown how the proximity and betweenness of network nodes can categorize them strongly. Betweenness refers to the differences and distances between clusters, and proximity refers to the close link inside the same cluster. In this regard, Horvitz H.R., Wang D.Y., and Sternberg P.W. stand out as reference authors.

### Citation analysis

There were “19,147” cited references, the front-ranking were Fire A (*Nature*, 1998), Lee RC (*Cell*, 1993), *C. elegans* Sequencing Consortium (*Science*, 1998), White JG (*Philosophical Transactions of the Royal Society B*, 1986), Reinhart BJ (*Nature*, 2000), Wightman B (*Cell*, 1993), Sulston JE (*Developmental Biology*, 1983), Epstein AC (*Cell*, 2001), Zou H (*Cell*, 1997), Kamath RS (*Nature*, 2003) (Table 3).

**Table 3:** Top 10 citation analysis of documents on *C. elegans*

Rank	First author	Year	Article title	Journal	Total citations (WOS)	Total citations per year (WOS)	Document type
1	Fıre A [16]	1998	Potent and Specific Genetic Interference By Double-Stranded RNA in *Caenorhabditis elegans*	*Nature*	10,944	405.33	Article
2	Lee RC [29]	1993	The *C. elegans* Heterochronic Gene Lin-4 Encodes Small RNAs with Antisense Complementarity to Lin-14	*Cell*	9158	286.19	Article
3	*C. elegans* Sequencing Consortium [32]	1998	Genome Sequence of the Nematode *C. elegans*: A Platform for Investigating Biology	*Science*	3985	147.59	Review
4	White JG [38]	1986	The Structure of the Nervous System of the Nematode *Caenorhabditis elegans*	*Philosophical Transactions of the Royal Society B*	3628	93.03	Article
5	Reinhart BJ [31]	2000	The 21-Nucleotide Let-7 RNA Regulates Developmental Timing in *Caenorhabditis elegans*	*Nature*	3331	133.24	Article
6	Wightman B [30]	1993	Posttranscriptional Regulation of the Heterochronic Gene Lin-14 by Lin-4 Mediates Temporal Pattern-Formation In *C. elegans*	*Cell*	3004	93.88	Article
7	Sulston JE [37]	1983	The Embryonic Cell Lineage of the Nematode *Caenorhabditis elegans*	*Developmental Biology*	2978	70.90	Article
8	Epstein AC [39]	2001	*C. elegans* Egl-9 And Mammalian Homologs Define A Family Oof Dioxygenases That Regulate HIF by Prolyl Hydroxylation	*Cell*	2683	111.79	Article
9	Zou H [43]	1997	Apaf-1, A Human Protein Homologous To *C. elegans* Ced-4, Participates in Cytochrome C-Dependent Activation of Caspase-3	*Cell*	2669	95.32	
10	Kamath RS [28]	2003	Systematic Functional Analysis of the *Caenorhabditis elegans* Genome Using RNAi	*Nature*	2648	120.36	Article

“Potent and Specific Genetic Interference by Double-Stranded RNA in *Caenorhabditis elegans*” [16] was the highest-cited reference in the field of *C. elegans*, with 10,944 citations. In this article, RNA was experimentally transfected into adult *C. elegans* cells and endogenous gene expression manipulation was observed. As a result, it has been shown that double-stranded RNA is much more effective in generating interference than using both strands separately. On the other hand, the article titled “Systematic Functional Analysis of the *Caenorhabditis elegans* Genome Using RNAi” [28] ranks last in the top 10 most cited articles in Table 3 with 2648 citations. According to the findings of this article, the function of ~ 86% of the 19,427 predicted genes of *C. elegans* was inhibited by RNAi interference, and mutant phenotypes were identified for 1722 genes. In addition, genes with similar functions have been shown to cluster in distinct, multi-megabase regions of individual chromosomes.

The second most cited study by Lee and colleagues in 1993 was titled “The *C-elegans* Heterochronic Gene Lin-4 Encodes Small RNAs with Antisense Complementarity to Lin-14” [29]. The researchers investigated the mechanism of action by negatively regulating the level of *lin-4*, lin-14 protein, which is required for the normal temporal control of various post-embryonic developmental events in *C. elegans*. They showed that all four *Caenorhabditis* clones obtained after cloning of the *C. elegans lin-4* locus functionally retained the *lin-4* null allele of *C. elegans* and that by directed mutagenesis of this region, *lin-4* does not encode a protein. They also identified approximately two small *lin-4* transcripts in *C. elegans* and showed that the *lin-14* mRNA contains sequences complementary to a repeated sequence element in the 3′ untranslated region (UTR). They also suggested that *lin-4* regulates *lin-14* translation through antisense RNA-RNA interaction. In another study conducted by the Wightman working group in the same year [30], it was shown that a temporal change in the lin-14 protein was produced post-transcriptionally by multiple elements in the lin-14 3′UTR, which is regulated by the heterochronic gene *lin-4.* In addition to these studies, another most cited study by Reinhart et al. [31], which is shown in Table 3, showed that sequential stage-specific expression of *lin-4* and *let-7* regulatory RNAs triggers transitions in the complement of heterochronic regulatory proteins to coordinate developmental timing.

One of the top 10 most cited articles on *C. elegans* research given in Table 3 was the review titled "Genome Sequence of the Nematode *C. elegans*: A Platform for Investigating Biology". This review of the genome sequence of the *C. elegans* nematode was conducted by the *C. elegans* Sequencing Consortium in 1998 [32], and this review became the third most cited article, with a total of 3985 citations according to 2023 records (Table 3). This review highlighted that the 97-megabase genomic sequence of the nematode *C. elegans* has more than 19,000 genes, with significant matches in other organisms for more than 40% of its predicted protein products. Additionally, they reported that the differential distribution of some repeats and highly conserved genes provided evidence for the regional organization of chromosomes.

Sydney Brenner, Robert Horvitz, and John Sulston’s discoveries regarding genetic regulation of organ development and programmed cell death have opened up truly new avenues for biological and medical research, and they were awarded the 2002 Nobel Prize in Physiology or Medicine [33]. As a result of the genetic analysis of *C. elegans*, especially as a result of studies involving Hortvitz, three genes, namely *ced-3*, *ced-4*, and *ced-9*, were identified that control the general apoptotic program [34, 35]. In light of the results of these studies, in the study carried out by Zou and colleagues in 1997, it was shown that Apaf-1, a human protein homologous to *C. elegans ced-4*, could lead to apoptosis by participating in the cytochrome c-dependent activation of caspase-3 [36].

Within the most cited articles listed in Table 3, the embryonic cell lineage of *C. elegans* was elucidated by Sulston and colleagues in 1983 [37], and the structure of the nervous system was elucidated by White and colleagues in 1986 [38]. Additionally, in 2001, Epstein and his colleagues focused on HIF, a transcriptional complex that plays a central role in mammalian oxygen homeostasis. By identifying a conserved HIF-VHL-prolyl hydroxylase pathway in *C. elegans*, they demonstrated that EGL-9 functions as a dioxygenase that regulates HIF via prolyl hydroxylation [39].

According to the general evaluation of the most cited articles from *C. elegans*-focused studies, elucidation of gene functions that play a critical role in *C. elegans* development, especially *lin-4* and *lin-14* functions, determination of mutant phenotypes through gene silencing with RNAi and their effects on *C. elegans* development as well as determining the genes that play a role in the development of *C. elegans*, determining the genes that play an important role in the mechanisms of apoptosis and hypoxia, and determining the functions and structural analysis of embryonic cell lineages and nervous systems have been the subject of interest of many researchers.

### Keywords and research area analysis

**Fig. 11:**
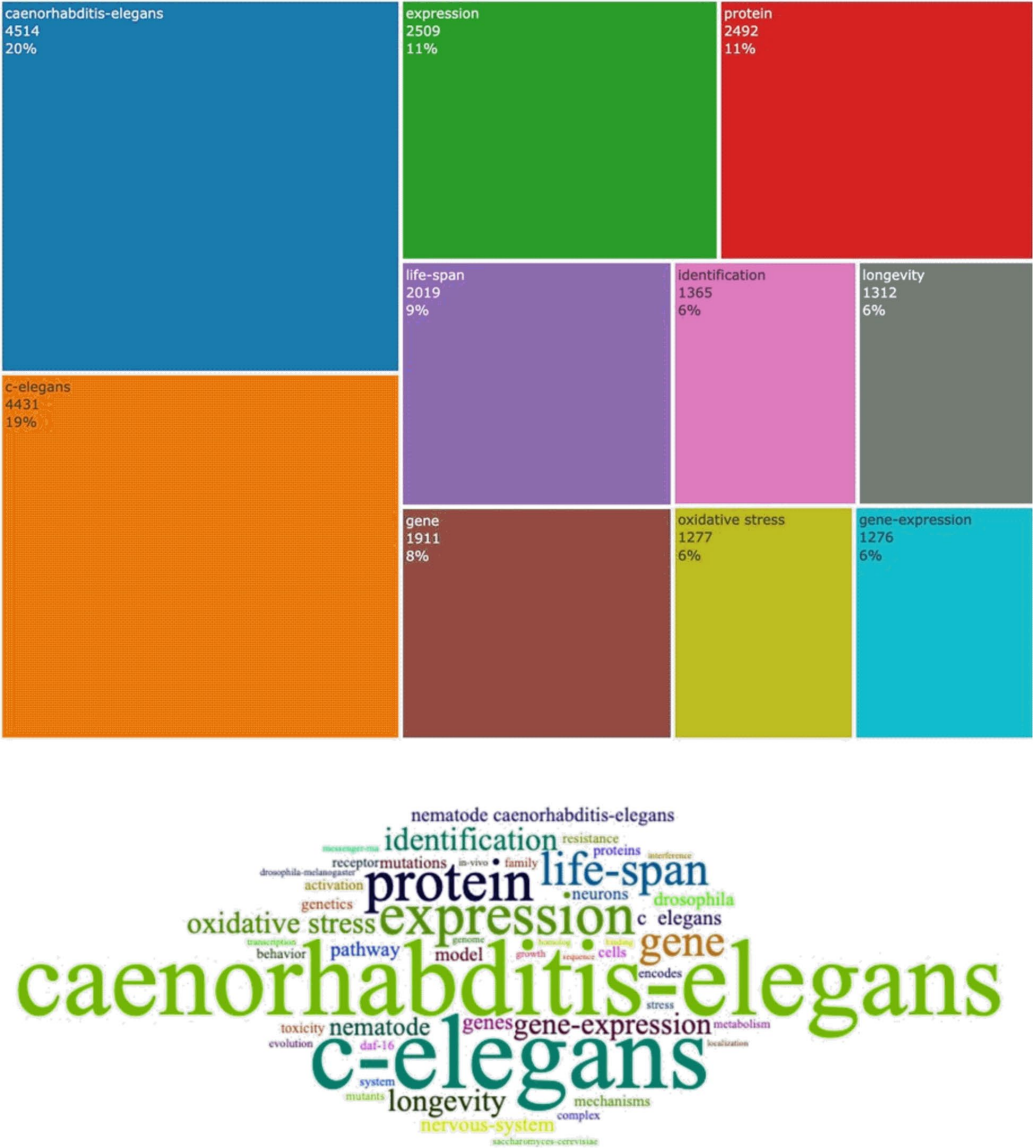
Treemap of top 10 keywords. Authors’ construct (2023) from the bibliometrix Package

We can mine and count the high-frequency keywords used by the authors of the investigated research papers using the package biblioshiny, filter the keywords with word frequencies equal to 10, and generate a word tree map (Fig. 11). In this regard, the most commonly used keywords in the research topic are *Caenorhabditis elegans*, *C. elegans*, expression, protein, lifespan, gene, identification, longevity, oxidative stress, and gene-expression accounting for 20%, 19% 11%, 11%, 9%, 8%, 6%, 6%, 6%, and 6% of the high-frequency keywords, respectively.

### Three-field plot

**Fig. 12:**
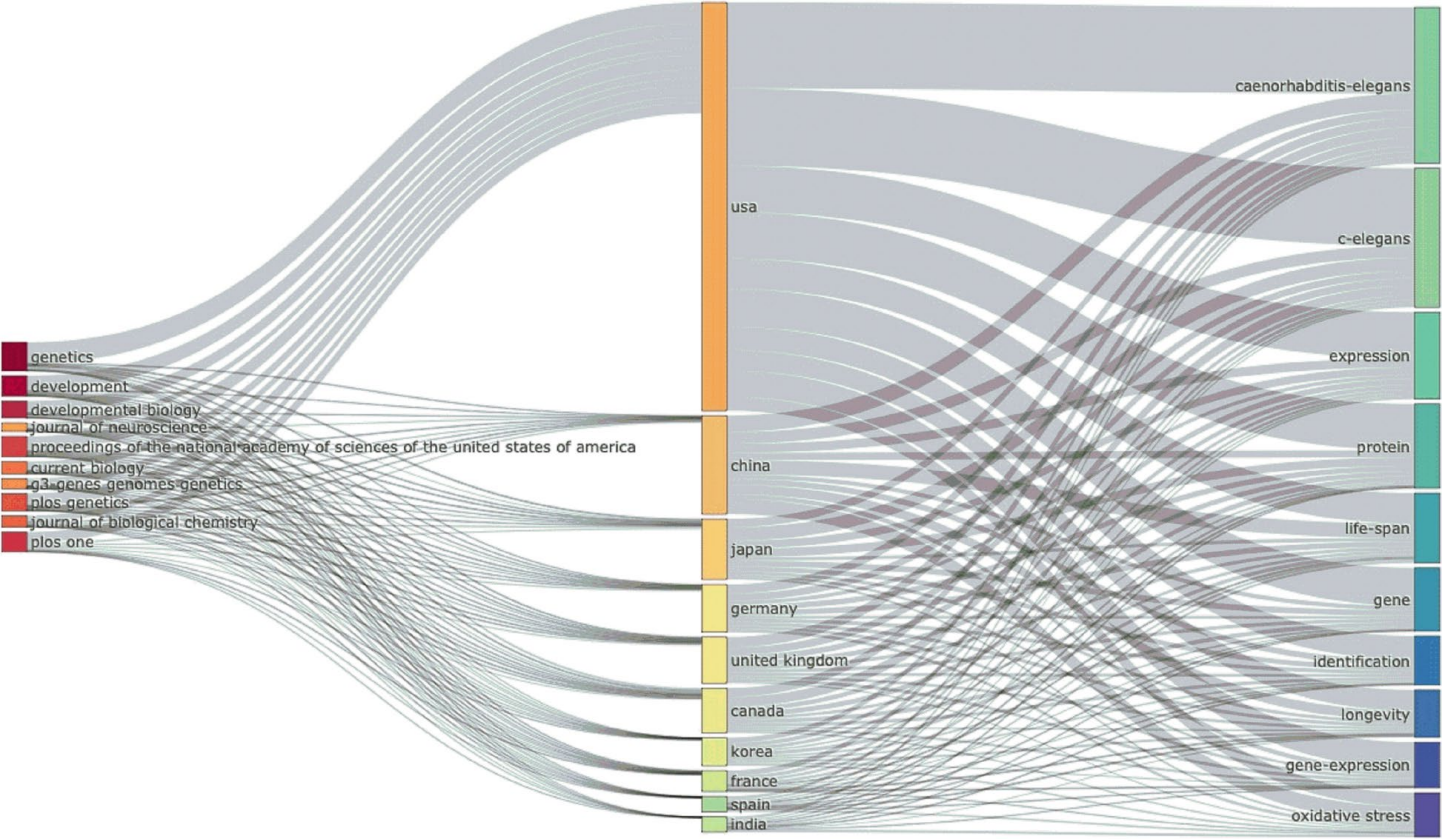
A three-field plot (Sankey diagram) of country, keyword, and journal of publication of the cited references for the ten most researched topics (created by Biblioshiny)

To obtain insight into the trends in publications, a 3-Field Plot Sankey Diagram (Fig. 12) was created with 10 items in each field based on the author’s country, author’s keywords, and source of publication. This map summarizes the relative importance of themes, the country doing the research, and the journals in which the work was published, with thicker rectangles representing greater frequency and numerous thick inflows and outflows representing more links. The linkages among sources (left), countries (middle), and author keywords (right) are studied using a three-field plot to determine which keywords are preferred by which countries and used by which sources. The top three countries (the USA, China, and Japan) have strong connections with the sources “Genetics” and “Development” and prefer to publish four keywords (*Caenorhabditis elegans*, *C. elegans*, expression, and protein). The block length in Fig. 12 reflects the degree of connectivity.

### Limitations

There are certain limitations to this study that are inherent in bibliometric studies. Because the data was gathered from WoSCC, publications indexed in other databases that could have been included were overlooked. Because of its enormous catalog, scientific influence, and comprehensive information for this type of study, this database is one of the most extensively utilized by academics for bibliometric analysis [40, 41]. Information was extracted using bibliometric techniques, which may have resulted in bias [42]. Furthermore, the analysis only included papers written in English.

The number of studies on *C. elegans* has steadily increased over the past 40 years, and the organism has continued to be the focus of academics and organizations. Despite the fact that numerous countries, companies, and academics were interested in the *C. elegans* research model, the active organizations and authors, particularly the top teams in this field, still lacked global collaboration.

## Conclusion

Bibliometric analysis, which differs from literature review and is based on a literature system such as the WoSCC database, is frequently used to evaluate current topics, research progress, and other related topics, as well as to develop future research directions and plans. Visual analysis allows the identification of key opinion leaders and future research directions; It reveals the network of research referrals as well as the collaborative link between organizations, nations, and authors, allowing the development of optimized future collaboration networks. This bibliometric analysis provides a global perspective on findings from *C. elegans* research and highlights the characteristics of 24,496 documents published in WoSCC from 1980 to 2023, comprising 20,322 individual studies and review articles on *C. elegans*. From 1980 to 2023, *C. elegans* research shows an exponential growth trend. Therefore, *C. elegans* is a topic that is receiving increasing attention and is likely to continue gaining momentum. There were 41,346 co-authors on this topic from 96 countries/regions; During this period, the USA, China, Japan, and the UK were the most productive regions. Horvitz H.R., Wang D.Y., and Sternberg P.W. are the most relevant authors, and the USA provides the greatest amount of scientific information on the topic (43% of articles). Additionally, the most dynamic journal is Genetics, while the most interesting journals include *Nature* and *Cell* for *C. elegans* researchers.
